# A Woman With Continuous, Involuntary, Unilateral Movement

**DOI:** 10.1016/j.acepjo.2025.100252

**Published:** 2025-10-08

**Authors:** Raymond Che, Suhani Patel, Michael Edwards, Michelle Wilson

**Affiliations:** 1 Department of Emergency Medicine, University of Kansas Medical Center, Kansas City, Kansas, USA; 2 Department of Emergency Medicine, University of Kansas Medical Center, Kansas City, Kansas, USA

**Keywords:** nonketotic hyperglycemic hemichorea, hemiballismus, diabetic striatopathy

## 1 Case Presentation

A 59-year-old woman with type 2 diabetes mellitus presented to the emergency department with 4 days of continuous, involuntary movements of her left arm and leg. She reported poor adherence to her diabetes medications and significant psychosocial stress. Vital signs were stable. Examination revealed continuous, irregular, choreiform movements of the left upper and lower extremities (Video 1) without other deficits. A noncontrast computed tomography scan of the head was obtained (Fig). Laboratory results revealed a blood glucose level of 420 mg/dL, negative ketones, and normal osmolality.

**Video 1:**
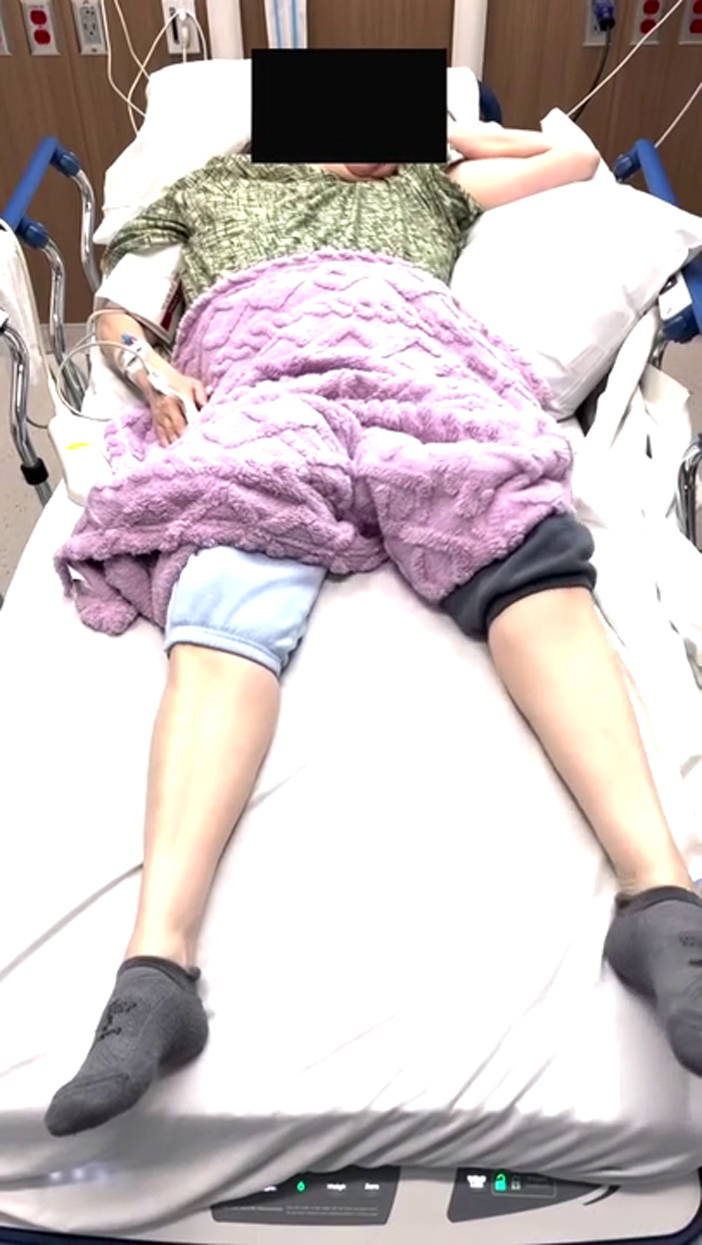
Continuous, involuntary, unilateral movement seen on examination.

**Figure:**
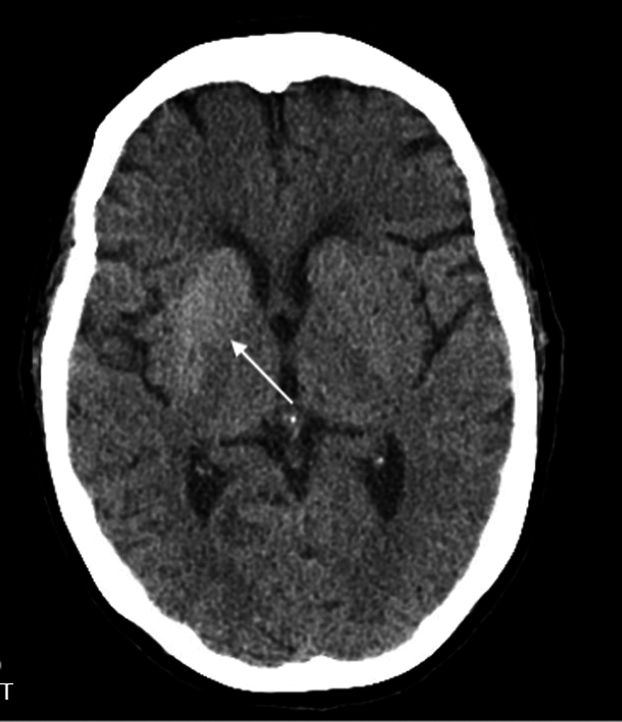
Diffuse right basal ganglia hyper-attenuation.

## 2 Diagnosis: Nonketotic Hyperglycemic Hemichorea Hemiballismus

Nonketotic hyperglycemic hemichorea hemiballismus, also called diabetic striatopathy, is a rare neurological complication of uncontrolled diabetes with a prevalence of approximately 1 in 100,000.^1^ It is the second most frequent cause of chorea after basal ganglia stroke.^2^

Diagnosis is made based on the presence of unilateral choreiform or ballistic movements in the setting of uncontrolled, often nonketotic, hyperglycemia, accompanied by characteristic imaging findings.^1^^,^^2^ Computed tomography typically demonstrates hyperdensity, whereas magnetic resonance imaging demonstrates T1 hyperintensity in the contralateral basal ganglia.^3^ The mechanism remains unclear.^1^

Treatment focuses on glycemic control. Around 25% of the patients improve with glucose correction alone, whereas others require medications such as haloperidol, tetrabenazine, or benzodiazepines to manage involuntary movements.^1^^,^^2^ Recovery varies from days to months, averaging 6 months.^1^ Some may experience only partial improvement, even after years, requiring long-term management. Our patient was started on fluids and an insulin drip, then admitted. She was discharged with improved glycemic control on Risperdal and as needed clonazepam for ongoing involuntary movements.

## Funding and Support

By *JACEP Open* policy, all authors are required to disclose any and all commercial, financial, and other relationships in any way related to the subject of this article as per ICMJE conflict of interest guidelines (see www.icmje.org). The authors have stated that no such relationships exist.

## Conflict of Interest

All authors have affirmed they have no conflicts of interest to declare.
